# Cancer Antigen-125 and Carcinoembryonic Antigen in Determining Malignancy Risk in Epithelial Ovarian Tumor: An Observational Study

**DOI:** 10.31729/jnma.9184

**Published:** 2025-08-31

**Authors:** Ranju Singh, Beemba Shakya

**Affiliations:** 1 Paropakar Maternity and Women’s Hospital, Thapathali, Kathmandu, Nepal

**Keywords:** *CA-125*, *CEA*, *epithelial ovarian cancer*

## Abstract

**Introduction:**

Epithelial ovarian cancers make up about 90% of ovarian malignancies and are often diagnosed late due to its vague symptoms. Cancer Antigen-125 (CA-125) and Carcinoembryonic Antigen (CEA), play a pivotal role in differentiating benign from malignant ovarian tumors and aid in assessing malignancy risk.

**Methods:**

The study was hospital-based cross-sectional study conducted at Paropakar Maternity and Women’s Hospital among 53 women diagnosed with ovarian tumors scheduled for surgery. Data were collected between July to December 2023. Non-epithelial tumors were excluded after obtaining final histopathology report. Preoperative CA-125 and CEA levels were correlated with epithelial ovarian tumors. Data analysis was performed using Statistical Package for Social Sciences software version 29.

**Results:**

Among 53 cases, 13 (33.30%) cases belonged to 40-49 years age group, while 5 (45.40%) cases of malignant tumors were in the 50–59 years age group. There were 5 (45.50%) cases of malignant tumors in women with parity two, while 11 (28.20%) cases of benign tumors in women with parity two. There were 39 (73.60%) benign cases, 3 (5.65%) borderline, and 11 (20.75%) malignant tumors. There were 36 (67.90%) serous tumors and 15 (28.30%) cases were mucinous tumors. Cancer Antigen-125 (>35 IU/mL) had high sensitivity 90.90%) and specificity (87.20%) for malignancy, while Carcinoembryonic Antigen (>3 ng/mL) had sensitivity 36.40% and high specificity 89.70% for malignancy.

**Conclusions:**

Cancer Antigen-125 showed high sensitivity and specificity in distinguishing malignant from benign epithelial ovarian tumors. Carcinoembryonic Antigen, while less sensitive, provided high specificity.

## INTRODUCTION

Ovarian neoplasm is the second most frequent gynecological cancer with a significant fatality rate worldwide.^1^ Epithelial ovarian tumors are heterogeneous neoplasms that are mainly divided into serous, mucinous, endometrioid, clear cell and transitional cell tumors based on the kind of cell they contain. Epithelial ovarian cancer (EOC) which is the malignant form of epithelial ovarian tumor constitutes 90% of all ovarian malignancies.^2^

Due to the lack of early specific symptoms, these tumors are often diagnosed at advanced stages, resulting in poor prognosis.^3^ Cancer Antigen-125 (CA-125) play a crucial role in differentiating benign from malignant ovarian tumors.^4^ Carcinoembryonic Antigen (CEA) is another most commonly used serum biomarkers for diagnosing solid malignancies including epithelial ovarian cancers.^5^

Because of the inadequate performance of CA-125 and CEA alone in the assessement of these ovarian tumors, this study aimed to analyse the importance of both of these tumor markers by correlating them with final histopathological report.

## METHODS

This study was a cross-sectional descriptive study conducted in 53 patients diagnosed as ovarian tumors admitted in the department of Obstetrics and Gynecology of Paropakar Maternity and Women’s Hospital (PMWH) from July 2023 to December 2023 following ethical approval from Institutional Review Board (IRB) (161/2080/81), National Academy of Medical Sciences (NAMS). A written informed consent was obtained from the patients who had ovarian tumor on clinical examination and/or radioimaging admitted in the hospital for surgery meeting the inclusion criteria for enrollment in the study. Patients with myoma, pregnancy, abdominal tuberculosis, acute peritonitis, pelvic masses from gastro-intestinal, breast origin and patients undergoing interval debulking surgery were excluded.

The convenient sampling method was used and the sample size was calculated using sensitivity with the formula,

n = Zα^2^ SN (1 - SN)

  (d^2^ × p)

= 1.96 × 1.96 × 0.87 (1-0.87)

  (0.1 × 0.1 × 0.9)

= 48

Considering 10% drop out rate, the sample size was 53.

Where,

Zα=1.96 taken at 95% of confidence interval

n = required sample size

p = prevalence of epithelial ovarian tumors according to previous comparable study is 90%^6^

SN = sensitivity of CA-125 according to previous comparable study is 87%^7^

d = 10% (Maximum tolerable error)

On the other hand, sample size with the formula using specificity is as follows,

n = Zα^2^ SP (1 - SP)

  (d^2^ × p)

= 1.96 × 1.96 × 0.95 (1-0.95)

  (0.1 × 0.1 × 0.9)

= 20

Considering 10% drop out rate, the target sample size was 22.

Where,

SP = specificity of CA-125 for Asian women according to previous comparable study is 95.60%^8^

Since the formula based on sensitivity yielded the larger sample size, the target sample size for this study was set at 53.

A detailed history and examination of patient was performed. Tumor markers CA-125 and CEA were sent in all patients as per hospital protocol. For analysis of serum CA-125 and CEA, patient’s blood sample of about 3-4 mL were taken in routine laboratory of PMWH in gel and clot activator tube (size 12x75 millimeters) during routine lab time from 10 am to 5 pm, within half hour to 45 minutes of sample collection. The gel tubes were centrifuged for 7-10 minutes at 2500-3000 RPM at room temperature. The serum thus obtained was processed in Beckman Assess-2 machine which used Chemiluminescence immunoassay (CLIA) method for detailed chemical analysis of level of CA-125 and CEA in patient’s serum. Serum CA-125 of ≤35 lU/mL and CEA of ≤3.5 ng/mL was taken as within the normal range. The reports were then recorded in proforma. Ultrasonography (USG) of abdomen and pelvis were undertaken. Other investigations like MRI, contrast enhanced CT (CECT) scans were also done as and when indicated.

Operative findings during surgery of all cases were documented. Immediately after opening the abdomen, ascitic fluid or 100ml peritoneal washing from each gutter was sent for cytological examination in a sterile syringe. It was made sure that the operated specimens (ovaries, tubes, uterus) or tissues were immersed in 10% formalin solution and were sent for histopathological analysis to the department of pathology at PMWH. The histopathological and peritoneal fluid cytology reports of corresponding patients were followed up in the pathology department of PMWH. The cases other than epithelial ovarian tumors were excluded from the study. The serum CA-125 and CEA values were correlated with the histopathological types of epithelial ovarian tumors. Data were entered and analyzed using the Statistical Package for the Social Sciences (SPSS) version 29 (IBM Corp., Armonk, NY, USA).

## RESULTS

A total of 80 patients with ovarian tumors were admitted during the study period. Of these, 5 patients were excluded (2 declined participation and 3 did not meet the inclusion criteria). Thus, 75 patients fulfilled the inclusion criteria. Following histopathological correlation, 22 cases were excluded, leaving 53 patients diagnosed with epithelial ovarian tumors.

In the benign group, 13 (33.30%) cases belonged to 40-49 years age group, followed by 10 (25.60%) cases in the group 20–29 years age group. There was 1 (33.33%) case with borderline tumors which belonged to 20–29 years age group and 2 (66.67%) cases belonged to 40–49 years age group. There were 5 (45.40%) cases of malignant tumors in the 50–59 years age group and 2 (18.20%) cases in the 40–49 years and 60 years age groups.

**Table 1:** Age and Parity Wise Distribution and Nature of Epithelial Ovarian Tumors (n=53).

	Benign n (%)	Borderline n(%)	Malignant n (%)
Age (years)			
10-19	1(2.60)	0(0)	0(0)
20-29	10(25.60)	1(33.33)	1(9.10)
30-39	7(17.90)	0(0.0)	1(9.10)
40-49	13(33.30)	2(66.67)	2(18.20)
50-59	3(7.70)	0(0)	5(45.40)
≥60	5(12.81)	0(0)	2(18.20)
Parity			
P0	9(23.10)	2(66.67)	3(27.20)
P1	4(10.20)	0(0)	0(0)
P2	11(28.20)	0(0)	5(45.50)
P3	5(12.80)	1(33.33)	2(18.20)
P4	6(15.40)	0(0)	1(9.10)

With respect to parity, 11 (28.20%) women with parity two had benign tumors, whereas 5 (45.50%) women with parity two had malignant tumors. Two (66.67%) cases of borderline tumors were seen in nulliparous women (Table 1).

**Figure 1:**
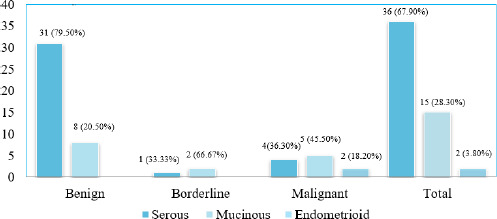
Histological types of epithelial ovarian tumors (n=53).

Among the 53 cases analyzed, 36 (67.90%) cases were serous tumors. There were 31 (79.50%) benign tumors of serous histologic type, 1 (33.33%) borderline tumors of serous histologic type, and 4 (36.30%) malignant tumors of serous in nature. Among 53 cases, 15 (28.30%) cases were mucinous tumors and 2 (3.80%) cases were endometroid tumors. (Figure 1).

There were 10 (90.90%) malignant cases were elevated levels of CA-125 > 35 IU/mL, while 5 (12.80%) benign cases showed elevated levels of CA-125 > 35 IU/m. For CA-125 ≤ 35 IU/mL, there were 1 (9.10%) malignant case and 34 (87.20%) benign cases. This corresponds to sensitivity of 90.90% and a specificity of 87.20% for CA-125 at the 35 IU/mL cutoff.

There were 4 (36.40%) malignant tumors were elevated levels of CEA > 3 ng/ mL and 4 (10.30%) benign cases had elevated levels of CEA > 3 ng/mL. 7 (63.60%) malignant tumours and 35 (89.70%) benign cases had CEA levels ≤ 3 ng/mL. This result corresponds to a sensitivity of 36.40% and a specificity of 89.70% (Table 2).

**Table 2:** Sensitivity and specificity of CA-125 and CEA in epithelial ovarian tumors (n=53).

Histopathology				
Tumor Marker	Malignant n (%)	Benign n (%)	Sensitiity	Specifiity
CA-125 > 35IU/mL	10(90.90)	5(12.80)	90.90	87.20
CA-125 < 35IU/mL	1(9.10)	34(87.20)		
CEA > 3ng/mL	4(36.40)	4(10.30)	36.40	89.70
CEA ≤ 3ng/mL	7(63.60)	35(89.70)		

## DISCUSSION

In this study, malignant epithelial ovarian tumors were observed even in younger patients aged 22-29 years, specifically in the form of mucinous cystadenocarcinoma. However, the majority of malignant epithelial ovarian tumors were more prevalent in women above 50 years of age. This was comparable to the study conducted by Jha et al., majority of benign tumors were seen in younger patients aged 20-49, while malignant tumors were more common in patients aged 50-59.^9^ Similar age distributions were observed by Amatya et al. and Karki et al.^10,11^

A considerable number of women with borderline (66.67%) and malignant (27.20%) ovarian tumors were nulliparous, echoing findings from previous studies by Jindal et al. and Saini et al. that associate nulliparity with a higher risk of ovarian cancer.^12,13^ In this study, among patients with malignant tumors, the P_2_ category (45.50%) was notably higher, which is comparable to the study conducted by Jindal et al.^12^, where the highest case distribution falls under parity 2 (28.07%), and 10.50% of ovarian malignancy patients were nulliparous, though increasing parity did not show a decrease in ovarian cancer.

In this study, benign epithelial ovarian tumors accounted for 39(73.60%) cases, followed by malignant 11 (20.75%) and borderline 3 (5.65%). Vaidya et al. in Kathmandu Medical College, Amatya et al. in Tribhuvan University, and Kanpurwala et al. in Mumbai found a similar distribution of tumors with benign (74%, 62.74%, 56.36%), borderline (8.23%, 5.88%, 10%), and malignant (17.72%, 31.37%, 33.63%) cases, respectively.^10,14,15^ The most common benign tumor in this study was serous (67.90%), contrasting with Kumari et al., where mucinous cystadenomas (35.90%) were most common.^16^ This could be due to the reason, as mentioned in the article, that delayed presentation in their study area might have favored the detection of larger, more symptomatic mucinous tumors earlier compared to other histological subtypes.

In contrast to this study, where the mucinous type is prevalent both in malignant category 5 (45.50%) and in borderline category 2 (66.67%), and serous tumors accounted for only 4 (36.30%) in the malignant category, the study conducted by Vaidya et al. showed that serous carcinoma was the most common malignancy 17 (60.71%) and the mucinous variety 10 (76.92%) was more prevalent in the borderline category.^14^ Similarly, in other studies conducted by Karki et al., Hota et al., Prakash et al., Rai et al. and Jha et al., among the malignant epithelial tumors, serous tumors (2.70%, 3%, 50%, 63.60% and 67.90% respectively), were most prevalent.^7,9,11,17,18^ This might be due to the difference in the sample size and duration. A smaller or shorter study, like the current study, might not capture the full variability in tumor types.

In this study, CA-125, with a cutoff value of > 35 IU/mL, showed high diagnostic accuracy for identifying malignant tumors resulting in a sensitivity of 90.90% and a specificity of 87.20%. This is comparable to the findings of Prakash et al., who reported a sensitivity of 87.18% and a specificity of 81.82% for predicting malignancy at the same cutoff value. ^7^ Similarly, Dongyong et al. found the sensitivity and specificity of CA-125 to be 87.70% and 10.80%, respectively, and of CEA to be 72.40% and 93.10%, respectively.^19^ In contrast, Wan et al. found CA-125 to be significantly elevated in patients with epithelial ovarian cancer; however, it had low sensitivity (59.02%) but high specificity (98%), while the sensitivity was increased (72.95%) when combined with determination of CEA.^20^ This might be due to the reason that a higher proportion of advanced-stage (Stage III-IV) cancers were enrolled in their study, which is associated with significantly elevated CA-125 levels. This might reduce sensitivity when including early-stage cancers where CA-125 levels are lower.

CEA, with a cutoff of 3ng/mL, had a lower sensitivity (36.40%) but a higher specificity (89.70%) in this study. The findings of this study align with those of Chakrabarti et al. and Lertkhachonsuk et al., who also reported a lower sensitivity (50% and 31.90%), respectively.^1,21^ However, CEA had a greater specificity (90.80%) in mucinous ovarian cancer in a study conducted by Lertkhachonsuk et al.^21^ In their studies, CEA was primarily associated with mucinous subtypes of ovarian cancer and was less effective in detecting serous tumors, which could explain the relatively low sensitivity observed. Wan et al. also reported that CEA, with lower sensitivity (51.64%) and higher specificity (94%), has limited utility as a broad marker for epithelial ovarian cancer.^16^ This is consistent with current study findings, where only 36.40% of malignant cases had elevated CEA levels.

The data suggest that while CA-125 is a highly sensitive and specific marker for identifying malignant ovarian tumors, CEA is more useful in confirming the absence of malignancy due to its high specificity, though it has lower sensitivity. This emphasizes the need to use these markers in combination, as CA-125 offers better overall diagnostic accuracy, while CEA provides additional specificity in certain cases.

The sample size for the CEA antigen was not calculated prior to study, and therefore it is uncertain whether the study was adequately powered for this analysis. Post hoc power analysis indicated that the statistical power for assessing the sensitivity and specificity of CEA in detecting malignancy in epithelial tumors was approximately 0.6. This suggests that the current sample size is insufficient, and a larger sample would be required to achieve adequate power.

## CONCLUSIONS

Serum levels of CA-125 were notably higher in malignant tumors compared to benign and borderline cases. CEA levels were elevated in malignant cases, particularly in mucinous tumors. In assessing the diagnostic accuracy, CA-125 showed high sensitivity and specificity in distinguishing malignant from benign epithelial ovarian tumors. CEA, while less sensitive, provided high specificity.

## Data Availability

The data are available from the corresponding author upon reasonable request.
